# Effects of garlic and lemon essential oils on performance, digestibility, plasma metabolite, and intestinal health in broilers under environmental heat stress

**DOI:** 10.1186/s12917-022-03530-y

**Published:** 2022-12-12

**Authors:** Ahmed M. Elbaz, Eman S. Ashmawy, Atif A. Salama, Abdel-Moneim Eid Abdel-Moneim, Faisal B. Badri, Hany A. Thabet

**Affiliations:** 1 Desert Research Center, Mataria, Cairo, Egypt; 2 Application Department, Nuclear Research Center, Atomic Energy Authority, 13759 Abu-Zaabal, Egypt; 3 Poultry Production Department, Faculty of Agriculture, Ain Shams University, Cairo, Egypt

**Keywords:** Broiler, Essential oils, Growth, Digestibility, Immunity, Antioxidant, Intestinal Health

## Abstract

**Background:**

Natural feed additives play an important role in poultry production due to their safety and potential properties as an antioxidant and antimicrobial, as well as a growth stimulant. The present research was designed to assess the influence of dietary supplementation of either garlic, lemon essential oil, or their mixture on performance, nutrient digestibility, plasma constituents, immunity, and oxidative status, as well as intestinal development assessed by microbiota—histomorphology development in broilers under environmental heat stress.

**Methods:**

A total of 480 broiler chicks (Ross 308) at one-day-old were randomly divided into four groups (120 chicks/ group). The control group received the basal diet (CON), while the other three groups received the basal diet supplemented with 200 mg/kg garlic essential oil (GEO), 200 mg/kg lemon essential oil (LEO), and their mixture (GLO) 200 mg/kg diet, respectively for 35 days.

**Results:**

The obtained results revealed that broilers fed essential oils as a mixture or individually had an improvement in average body weight, feed conversion ratio, carcass dressing, and an increase in digestive enzymes activities compared to the control group, furthermore, there was a reduction in the mortality rate and abdominal fat content. Adding essential oils as a mixture or individually led to a decrease in (*P* < 0.05) blood plasma triglycerides, cholesterol, low-density lipoprotein, and an increase in high-density lipoprotein. Broilers fed diets supplemented with essential oils as a mixture or individually had higher values of superoxide dismutase and glutathione peroxidase; while plasma malondialdehyde was lower (*P* < 0.05) compared to the control diet. Moreover, there was a significant enhancement in intestinal microbial content, and intestinal histological status of chickens fed with essential oils.

**Conclusions:**

Conclusively, including the mixture of essential oils improved performance, nutrient digestibility, and digestive enzymes activities. It also enhanced immunity, antioxidant state, and lipid profile, and gut microbiota— histomorphology in broilers. It was proposed that the broilers diet be supplemented with a mixture of essential oils to a mitigation of the effects of heat stress.

## Background

Recently, the poultry industry has been one of the largest economies in the world, especially after developing countries tended to support this industry to increase protein per capita. However, the poultry industry suffers from many challenges, including the high costs of feed ingredients, epidemics, and environmental challenges, the most important of which is global warming. The higher temperature ambiance leads to a negative impact on bird performance, and thus great economic losses for the poultry industry. The great interest of the nutritionist is the use of feed additives, which are an essential and influential part of the feed composition to enhance the performance and health of the bird [1]. Feed additives are used to alleviate heat stress concerns and as an effective antibiotics alternative such as probiotics, vitamins, etc. [2, 3]. There has been great interest in exploring products extracted from aromatic medicinal herbs and spices as antibiotics alternatives and immune enhancers. Many beneficial compounds in medicinal plants have been revealed to have an important role as an anti-inflammatory, antimicrobial [4], antioxidant, and enzyme production stimulus [5], which improves the efficiency of feed utilization.

Garlic (Allium sativum) is a perennial flowering plant utilized in herbal remedies, seasoning, etc. Garlic contains a high percentage of oil, which consists of featured organ sulfur compounds (allicin and diallyl sulfide) that act as anti-inflammatory, antioxidants, anti-atherosclerotic, and immune-modulation activities [1, 6]. In addition, it showed benefits when added to poultry feed in the form of powder, which led to an enhancement in growth, blood characteristics, and intestinal condition, mitigating the harmful effects of heat stress [2, 7]. Lemon (Citrus Limon) is a fruit rich in vitamin C, vitamin A and flavonoids, furthermore, it is infused with calcium, potassium, thiamine, pantothenic acid, and beta-carotene, as compounds that give several health benefits. Lemon essential oil is known for its content of some phenolic compounds which act as an antioxidant, immunomodulatory, antimicrobial, and digestive stimulant [8]. Lemon essential oil can reduces the negative effect of heat stress due to its content of vitamin C [9]. Earlier studies indicated that adding some lemon products to broiler diets can improve overall performance via reduce the negative effect of harmful bacteria and oxidative stress [10]. Previous studies results showed that feeding broilers a diet containing either garlic or lemongrass essential oil concentration of 200 mg/kg improved growth performance, and lipid profile as well as enhancing immunity and antioxidant status [5, 11]. This study aims to investigate the possible role of the inclusion of garlic, lemon essential oils, or their combination to mitigate the adverse effects of heat stress on performance, nutrient digestion, plasma lipids, oxidative status, and intestinal development in broilers that are exposed to environmental heat stress (hot climate).

## Results

### Growth performance and Carcass traits

The results showed that adding either garlic or lemon essential oil or both combined in the diet improved the growth performance and carcass traits of broiler chickens, as shown in Table 1. Average body weight (ABW), average feed intake (AFI), and feed conversion ratio (FCR) were not affected (*P* < 0.05) by the experimental treatments during the first 14 days. Nevertheless, the addition of essential oils led to a significant improvement in ABW and FCR, with a slight increase (*P* < 0.05) in the FI during different stages. There was a significant increase in ABW and a significant decrease in FCR in the GEO, LEO, and GLO groups compared to the CON group. In addition, there was an increase (*P* < 0.05) in the AFI in the GLO and LEO groups than those in the GEO and CON groups during the overall period. Furthermore, the mortality rate decreased and the European Production Efficiency Factor (EPEF) increased (*P* < 0.05) with the addition of essential oils to the diet. The carcass dressing and small intestine weight increased, meanwhile the abdominal fat content decreased (*P* < 0.05) in the essential oil-fed groups compared to the control group. The relative weight of the liver was not affected (*P* < 0.05) by the experimental treatments.

**Table 1:** Effect of dietary supplementation of essential oils on the performance of broilers at 35 days of age

	CON	GEO	LEO	GLO	SEM	*P*- value
**Growth performance**						
**0–14 day**						
ABW (g)	351	348	357	353	1.094	0.306
AFI (g)	386	379	385	384	0.988	0.197
FCR	1.10	1.09	1.08	1.09	0.027	0.240
**15–28 day**						
ABW (g)	832^b^	853^ab^	869^ab^	891^a^	3.421	0.011
AFI (g)	1376^b^	1401^ab^	1429^a^	1452^a^	8.133	0.026
FCR	1.654^b^	1.641^b^	1.642^b^	1.628^a^	0.080	0.040
**29–35 day**						
ABW (g)	559^c^	608^b^	635^a^	632^a^	5.217	0.018
AFI (g)	1412	1405	1423	1391	13.08	0.165
FCR	2.526^a^	2.311^b^	2.233^b^	2.201^c^	1.006	0.001
**0–35 day**						
ABW (g)	1740^c^	1811^b^	1862^a^	1875^a^	15.16	< 0.001
AFI (g)	3171^b^	3184^b^	3233^a^	3224^a^	29.07	0.038
FCR	1.821^a^	1.760^b^	1.737^bc^	1.718^c^	0.921	< 0.001
Mortality (%)	7.50	5.83	5.00	5.00	-	-
EPEF	252	277	290	296	-	-
**Carcass characteristics**						
Dressing	74.15^c^	75.36^b^	75.54^b^	77.80^a^	2.611	0.017
Liver	3.04	2.97	3.11	3.07	0.305	0.708
Abd. fat	2.57^a^	2.39^a^	2.45^a^	2.03^b^	1.244	0.022

Different superscript letters in the same row indicate significant differences (p < .05)

*CON* Control group, *GEO* Garlic essential oil group, *LEO* Lemon essential oil group, *GLO* Garlic with lemon essential oil group, *ABW* Average body weight, *AFI* Average feed intake, *FCR* Feed conversion ratio, *EPEF* European Production Efficiency Factor, *Abd. Fat*; Abdominal fat

### Nutrient digestibility and digestive enzymes

Garlic, and lemon essential oil, individually or as a mixture supplementation increased (*P* < 0.05) the content of dry matter and crude protein digestibility at 35 days (Table 2). There were no significant differences between the experimental groups regarding ether extract digestibility. The essential oil-supplemented diet increased (*P* < 0.05) the trypsin, lipase, and amylase activity in the ileum of broilers (Table 2). However, the activity of trypsin and lipase of broilers was observed to significantly increase (*P* < 0.05) by feeding GLO and GEO compared to the LEO and control groups. While the activity of amylase significantly increased by feeding on the GEO group compared to the other groups.

**Table 2:** Effect of dietary supplementation of essential oils on nutrient digestibility (%) and digestive enzymes activity (U/ml) of broilers at 35 days of age

	CON	GEO	LEO	GLO	SEM	*P*- value
**Nutrient digestibility (%)**						
Dry matter	70.8^b^	73.5^a^	74.0^a^	74.6^a^	1.033	0.013
Ether extract	72.6	71.9	72.3	73.0	2.411	0.102
Crude protein	68.1b	70.3^ab^	72.1^a^	72.8^a^	0.883	0.010
**Digestive enzymes activity (U/ml)**						
Amylase	3.18^c^	6.52^a^	4.32^b^	5.41^ab^	0.716	0.007
Trypsin	21.61^b^	22.08^b^	20.95^b^	23.91^a^	1.128	0.034
Lipase	9.07^b^	13.22^a^	9.51^b^	13.64^a^	0.095	< 0.001

Different superscript letters in the same row indicate significant differences (*p* < .05)

*CON* Control group, *GEO* Garlic essential oil group, *LEO* Lemon essential oil group, *GLO* Garlic with lemon essential oil group

### Plasma constituents

Plasma triglycerides, cholesterol, and LDL levels decreased in broilers fed garlic (GEO), lemon essential oil (LEO), or their mixture (GLO) compared to the control group, as shown in Table 3. However, there were no significant differences in the total protein, albumin, globulin, A/G ratio, creatinine, and urea levels of the broiler plasma (*P* < 0.05) among experimental groups. However, there was a slight numerical increase (P = 0.065) in total protein and the A/G ratio with the addition of essential oils compared to the control group.

**Table 3:** Effect of dietary supplementation of essential oils on the lipid profile, protein profile, and kidney function of broilers at 35 days of age

	CON	GEO	LEO	GLO	SEM	*P*- value
**Lipid profile(mg dl** ^**−1**^ **)**						
Triglycerides	224^a^	203^b^	186^c^	181^c^	1.006	0.011
Cholesterol	243^a^	192^b^	175^b^	180^b^	3.421	0.003
HDL	60.7^c^	75.2^b^	82.7^a^	86.6^a^	1.550	0.024
LDL	161^a^	108^c^	127^b^	105^c^	2.979	0.037
**Protein profile and kidney function**						
Total protein(g dl^−1^)	3.21	3.45	3.22	3.28	0.711	0.125
Albumin (g dl^−1^)	2.03	1.97	2.13	2.04	0.699	0.080
Globulin (g dl − 1)	1.18	1.48	1.09	1.24	0.095	0.121
A / G ratio	1.72	1.33	1.95	1.65	0.087	0.065
Creatinine (mg.dl^−1^)	0.52	0.57	0.60	0.55	0.041	0.203
Urea(mg.dl^−1^)	0.64	0.61	0.63	0.67	0.080	0.110

Different superscript letters in the same row indicate significant differences (*p* < .05)

*CON* Control group, *GEO* Garlic essential oil group, *LEO* Lemon essential oil group, *GLO* Garlic with lemon essential oil group, *HDL*, High-density lipoprotein, *LDL*, Low-density lipoprotein

### Immune organ weight and humoral immunity

The effect of essential oils supplementation on the immune response, immune organs, and humoral immunity are summarized in Table 4. Adding essential oils individually or as a mixture significantly increased the relative weight of the bursa of Fabricius (*P* < 0.05). Furthermore, the results showed that the serum antibody titer against NDV significantly increased in LEO, GEO, and GLO groups compared to the control group. Broilers fed dietary supplementation of COE had the highest (P < 0.05) antibody level against NDV. No significant changes were noticed in relative weights of spleen, thymus, and antibody titer against AIV between dietary treatments (*P* < 0.05).

**Table 4:** Effect of dietary supplementation of essential oils on the immune organ weight (%) and humoral immunity of broilers at 35 days of age

	CON	GEO	LEO	GLO	SEM	*P*- value
**Immune organs**						
Spleen	1.16	1.20	1.19	1.13	0.172	0.133
Thymus	3.04	3.27	3.35	3.19	0.091	0.107
Bursa of Fabricius	2.51^c^	3.11^b^	3.60^a^	3.47^ab^	0.070	0.001
**Humoral immune**						
NDV	5.07^c^	5.53^b^	5.41^b^	5.84^a^	0.161	0.020
AIV	2.81	2.95	3.03	2.98	0.118	0.095

Different superscript letters in the same row indicate significant differences (*p* < .05)

*CON* Control group, *GEO* Garlic essential oil group, *LEO* Lemon essential oil group, *GLO* Garlic with lemon essential oil group, *NDV* Newcastle disease virus, *AIV* Avian influenza virus (H9N1)

### Antioxidant and liver enzyme

Glutathione peroxidase (GPx), and superoxide dismutase (SOD) levels (*P* < 0.05) were elevated in the LEO, GLO, and GEO groups compared to the control group as shown in Table 5. The malondialdehyde level (MDA) of plasma in broilers fed diets with the LEO, GLO, and GEO was lower than it is in the control group. However, liver enzyme activity, such as alanine aminotransferase (ALT), and aspartate aminotransferase (AST), were not affected by the experimental groups (*P* < 0.05).

**Table 5:** Effect of dietary supplementation essential oils on antioxidant and liver enzymes of broilers at 35 days of age

	CON	GEO	LEO	GLO	SEM	*P*- value
**Antioxidant enzymes**						
MDA (nmol ml^−1^)	1.431^a^	0.915^c^	1.064^b^	0.805^d^	0.620	0.001
SOD (U ml^−1^)	120.7^c^	139.4^a^	131.1^b^	140.2^a^	1.085	0.016
GPx (U ml^−1^)	21.13^c^	33.70^a^	29.44^b^	32.08^ab^	1.316	< 0.001
**Liver enzymes**						
ALT (U L^−1^)	26.70	27.12	25.64	26.51	1.299	0.117
AST (U L^−1^)	209.1	198.7	211.4	206.3	0.705	0.095

Different superscript letters in the same row indicate significant differences (*p* < .05)

*CON* Control group, *GEO* Garlic essential oil group, *LEO* Lemon essential oil group, *GLO* Garlic with lemon essential oil group, *MDA* Malondialdehyde, *GPx* Glutathione peroxidase, *SOD* Superoxide dismutase, *ALT* Alanine aminotransferase, *AST* Aspartate aminotransferase

### Microbial count and Intestinal Development

Supplementing the diet with essential oils increased cecal *Lactobacillus* and reduced *E. coli* count at 35 d of age compared to control broilers (P < 0.05), meanwhile there were no significant differences in total lactic acid bacteria count (Table 6). The GLO and LEO groups had higher cecal *Lactobacillus*, and lower *E. coli* count compared to the rest of the groups (*P* < 0.05). Broilers-fed diets supplemented with essential oils had higher (P < 0.05) VH, and lower CD than the control group, as shown in Table 6. Villus height (VH) and crypt depth (CD) in the GLO and LEO groups were higher (*P* < 0.05) than it is in the GEO and CON groups. However, the GEO group had higher VH and lower CD than the other groups. The VH to CD ratio didn't significantly change among experimental groups (P < 0.05).

**Table 6:** Effect of dietary supplementation essential oils on microbial enumeration (Log^10^ CFU g^− 1^), and histomorphological (µm), of broilers at 35 days of age

	CON	GEO	LEO	GLO	SEM	*P*- value
**Antioxidant enzymes**						
MDA (nmol ml^−1^)	1.431^a^	0.915^c^	1.064^b^	0.805^d^	0.620	0.001
SOD (U ml^−1^)	120.7^c^	139.4^a^	131.1^b^	140.2^a^	1.085	0.016
GPx (U ml^−1^)	21.13^c^	33.70^a^	29.44^b^	32.08^ab^	1.316	< 0.001
**Liver enzymes**						
ALT (U L^−1^)	26.70	27.12	25.64	26.51	1.299	0.117
AST (U L^−1^)	209.1	198.7	211.4	206.3	0.705	0.095

Different superscript letters in the same row indicate significant differences (*p* < .05)

*CON* Control group, *GEO* Garlic essential oil group, *LEO* Lemon essential oil group, *GLO* Garlic with lemon essential oil group, *MDA* Malondialdehyde, *GPx* Glutathione peroxidase, *SOD* Superoxide dismutase, *ALT* Alanine aminotransferase, *AST* Aspartate aminotransferase

## Discussion

Several studies have investigated nutritional supplements to reduce the harmful effects of heat stress on the bird. During hot weather, broiler chickens are affected by a disorder in the oxidation system and the microbial composition of the intestines in addition to changes in the mechanisms of metabolism, this makes the bird more susceptible to deterioration of the bird’s performance and high mortality. Wherefore, in our research paper, we focused on clarifying the potential benefits of adding essential oils to broiler feed which are exposed to high-temperature conditions (hot climate) on productive performance, physiological changes, and intestine developments.

The present study demonstrated that essential oil supplements enhanced performance by enhancing antioxidative and digestive enzymes, improving nutrient digestibility, and intestinal health [12, 13]. The results also showed a decrease in the productive performance of the control group as a result of exposure to high temperatures, this may be due to the deficiency in feed intake and feed efficiency as a result of changes in metabolism, the same reported by Sohail et al. [14]. Our results were identical to those of Shakeri et al. [15] who reported a decrease in the productive efficiency of broilers exposed to heat stress. The results of the statistical analysis showed a decrease in the FI for the control group as a negative effect of high temperature (hot climate), which appeared in the high FCR and low ABW. Interestingly, the addition of essential oil alone or in combination had a positive effect on the productive performance by improving the growth performance, feed conversion ratio, and decreasing mortality rate. These results agree with other results that have shown broiler chickens under environmental heat stress that feed on essential oils such as oregano [16], and garlic [2] led to growth performance improvements. Similarly, Hong et al. [10] observed that adding essential oils has resulted in an improvement in growth performance. The reduced negative effect of high temperature on the productive performance of broiler chickens fed a diet containing essential oil may be through the effect of essential oils on the activity of antioxidants, digestive enzyme secretion, and antimicrobial activities, as indicated by Alagawany et al. [5]; Patra [17]. Our results also indicated a slight increase in FI with the addition of essential oils, especially with the addition of lemon essential oil (LEO and GLO). Saleh et al. [18] reported that supplementation of essential oils to broiler feed significantly increased FI. However, Ocak et al. [19] observed that supplementation of a mixture of essential oils to broiler chickens reduced FI. The discrepancy in the other studies results on the feed intake may be due to the type of essential oil extracted, the method of extraction, or the added quantity and its effect on the gut health and the oxidative state of the birds.

In this study, our results showed a significant increase in the carcass dressing and small intestine weight and a decrease in the content of abdominal fat. Similar results were reported by Alcicek, et al. [20] who reported that essential oils as feed additives improved carcass yield. Several studies have reported that some of the most important advantages of feed additives are being used as alternatives to antibiotics or as a booster to take advantage of the nutrients digestibility in the diets to improve carcass characteristics [2, 5]. The increase in carcass dressing in broilers fed essential oil may be due to the effect of essential oils on the activity of the digestive enzyme, and antimicrobial activities, which is reflected in improved carcass dressing. Furthermore, there was a significant increase in the relative weight of the small intestine in chickens fed essential oils compared to chickens fed the control diet. Same findings with Issa and Omar. [21]; Elbaz [22]. Moreover, the results showed less abdominal fat in chickens fed GLO than those fed GEO, LEO, and control diet. This represents an economic benefit in the poultry industry, where abdominal fat is considered a waste in the slaughterhouse [23] because it reduces carcass yield. Our results showed a significant improvement in CP and DM digestibility in birds fed essential oils compared to control-fed birds. The GLO and LEO significantly increased CP digestibility compared to GEO and CON groups, while the digestibility of DM in broilers fed on either essential oils alone or as a mixture increased compared to control. It is interesting to find a pungent smell to adding garlic (GEO and GLO groups), which may stimulate the secretion of saliva and gastric juices in broiler chickens [13]. The improvement in nutrient digestion may also be due to the effects of herbaceous plant extract in modifying the microbial content of the bird's gut, which enhances nutrient metabolism [24, 25]. On the other hand, many studies showed that the addition of essential oils enhanced the activity of digestive enzymes and balanced the gut microbial ecosystem, which in turn improved the ability to digest nutrients [26, 27]; also is confirmed by our results mentioned. There was a significant increase in the activity of digestive enzymes with the addition of essential oils to broiler feed in this study. The activity of amylase and lipase increased in birds fed LEO and GEO, while trypsin activity increased in birds fed GLO compared to the other groups, these results were in agreement with the findings by Xu et al. [26]. Some studies showed that the activity of some other enzymes improved by essential oil addition [28]. However, other studies have shown that the addition of essential oils did not enhance nutrient digestion [29, 30]. The difference in results may be due to the types of essential oils used or the amount of addition, as some oils had a harmful (irritating) effect on the wall of the intestinal lining, which leads to some inflammation [25], resulting in reduced utilization of nutrients. Therefore, it is necessary to determine the type and quantity of essential oils used for broiler chickens.

Biochemical changes in broiler plasma are due to the physiological outcomes of the feed additives, which indicate the bird's health status. In the current study, dietary supplementation of essential oils decreased the triglycerides, cholesterol, and LDL concentration and increased HDL concentration in plasma compared to the control group. Our results were consistent with previous investigations, by Prasad et al. [29]. The hypocholesterolemic in chicken-fed garlic can be explained by the fact that it contains some active compounds that reduce the liver activity of cholesterogenic and lipogenic enzymes such as a malic enzyme, fatty acid synthase, and glucose-6-phosphatase dehydrogenase [21]. Both GEO and LEO decreased LDL levels and increased HDL levels compared to the levels in the control group birds. The change in LDL and HDL levels resulting from the effect of essential oils antioxidants and anti-peroxide on liver activity (inhibiting hepatic fatty acid synthase), which affects the biosynthesis of cholesterol [30]. These results show that the addition of natural essential oils has an effective lipid-enhancing effect by reducing cholesterol and increasing lipid stability, thus reducing lipid oxidation problems during heat stress. Studying the effect of dietary supplements on serum protein is necessary because it is an indication of chronic or acute inflammatory conditions, especially when the bird is exposed to stress. The decrease in albumin and an increase in globulins, the decrease in the A: G ratio is indicative of inflammation, aspergillosis, nephropathies, and liver failure [31]. Our results showed a numerical increase in the serum protein, albumin, and globulin, as well as the A: G ratio *(p = 0.065)* in chickens fed on experimental supplements compared to chickens fed on the control. Similar results were reported by Alzawqari et al. [32] reported that dietary supplementation of lemon essential oils increased the total protein, and albumin levels, and reduced liver enzyme activities. The same findings were with [33]. The effect of adding essential oils in decreasing levels of liver enzymes in broilers may be due to their ability to repair hepatic injury or restore the cellular permeability that can be caused by cytotoxic compounds or via a cytoprotective influence due to its phenolic components Tiwari et al. [34]. The reason for the absence of a significant influence on the serum protein may be due to the quality of the oil extraction, the composition of the diet, the oil concentration, or the conditions of the current experiment.

The current study focused on changes in the immune systems that reflect the cellular and mixed immune status (the function of the immune system), especially since many studies have proven the impact of exposure to heat stress on the relative weights of the immune systems of chickens [35, 36]. This explains the reason for the low relative weight of the bursa of Fabricius in the control group, which may be due to the increase in the level of corticosterone in heat-stressed chickens, which prevents the growth of immune organs or as a result of the lack of feed intake, which causes a lack of nutrients necessary for the proper development of immune organs [37]. Many studies reported that essential oils have extensive potential antibacterial and anti-inflammatory activity [4, 6], So it was expected that the level of immune response would improve after feeding certain plant essential oils. The immune organs of poultry are the main organs that secrete many kinds of immune activity factors, and their status is exceedingly related to immune function [38, 39]. Usually, the relative weight of the immune organs is used to assess the immune status of the bird, as it is represented in the largest weight and a stronger immune function to some extent [38]. Our finding showed that a significant improvement in the immune response of birds exposed to high temperatures fed with the addition of essential oils. The relative weight of the bursa of Fabricius and the antibodies against NDV increased in chickens fed diet containing the essential oils. The results were consistent with previous investigations, by Hanieh et al. [40] which illustrated that garlic supplementation in broiler chicken increases the relative weights of the immune organs. Enhancement in the immune response and disease resistance by using bio-additives in the diet has been reported in several studies [2, 41], which is consistent with the results of this study. Nidaullah et al. [42] concluded that adding herbs plays a vital role as an immunostimulant against NDV and infectious bursal disease. It is suggested that essential oil modulation impact on gut microbes could play a beneficial role in the development of the immune system [43], The noticeable improvement in immunity may be due to the properties of biologically active compounds in essential oils as antimicrobial [6], antioxidant [8], and anti-inflammatory [4], which help in providing nutrients necessary for the development of the immune system by promoting the proliferation of lymphocytes in the primary immune organs and improving intestinal integrity [44], Thus stimulating the production of antibodies (immunoglobulin, as IgG, IgM, and IgA). The increase in the level of immunoglobulins is associated with the relative weight of the immune organs [38]. We concluded that the addition of these essential oils has a potent impact on immunomodulation in chickens by providing more nutrients needed to synthesize antibodies and develop the immune organs.

One of the most important problems of heat stress on the bird is oxidative stress. Oxidative stress results from an imbalance between oxidation and reduction in the cell, which causes significant damage to the cell and the health of the bird. The first line of defense against oxidative stress is the antioxidant enzymes that are responsible for preventing free radical attacks on biomembranes [45]. Medicinal, aromatic plants or their essential oils have different biological activities, the most influential of which are natural antioxidants, which have therapeutic effects that enhance the health of the birds [46, 47]. Therefore, it was necessary to study the effect of adding essential oils on the antioxidant enzymes of chickens exposed to high temperatures. The results of the current study indicated that the addition of essential oils decreased MDA levels, and increased SOD and GPx levels compared to the control group; this indicates that the addition of essential oils increased the ability of broilers to remove oxygen free radicals. The results were consistent with previous investigations, Chen et al. [48]; Lin et al. [49] which illustrated that serum GSH-Px and SOD activities significantly increased with the addition of natural plants to the diet. The current study showed a significant decrease in MDA level with the addition of essential oils, and this indicates a lack of oxygen production, which confirms that the addition of essential oils reduced the harmful effect of heat stress. Al-Sagheer et al. [41] clarified that lemongrass oil supplementation enhanced antioxidant indices by reducing MDA content.

The digestive system contains about 70–80% of immune cells in the body and a large group of gut microbes, which play a major role in the bird's immunity. The intestine is one of the most sensitive organs to environmental stress, which leads to great damage to the health of the bird. It is recognized that stress influences the composition, structure, and activity of the gut microbial community [50]. Feed additives play an important role in modifying the microbial content of the intestine, especially during periods of stress. In this study, total lactic acid bacteria counts were not significantly affected by the treatments, while *E. Coli* counts significantly increased, and *Lactobacillus* counts significantly decreased in birds fed the basal diet compared with those receiving the essential oil diets. Several studies have shown some modifications in the microbial content of chickens exposed to heat stress, including higher counts of *Clostridium* and *E. coli* and lower counts of Lactobacillus [51, 52]. This explains the harmful effect of heat stress on the microbial composition of the intestine, which negatively influences growth performance. This can be explained by the impact of heat stress via reducing feed intake and increasing water consumption, which affects the provision of nutrients to the gut microbes, and changes the intestinal environment (digesta viscosity and patterns of secretory activity), leading to inflammation and damage in the mucous membrane as a result of oxidative stress and toxins penetrating the intestinal barrier [53, 54]. Our results showed a significant improvement in the microbial community structure with the addition of essential oils, as the number of *Lactobacillus* increased and *E. coli* decreased. This result is consistent with previous some study reported that essential oils supplementation has a positive effect on gut microbiota [55, 56]. Our results show the important role of essential oils as a modifier of intestinal microbes, which can be added as an alternative to antibiotics, and act as an anti-inflammatory and antimicrobial leading to an improvement in gut health with a concomitant enhancement in growth performance.

Histomorphological parameters, such as VH and CD, are an indicator of the absorptive capacity of the small intestine and evaluating intestinal health [55]. The present study demonstrated that the addition of essential oils enhanced VH and CD of the ileum. It has been reported that the supplementation of essential oils enhanced VH and CD [10]. However, Alagawany et al. [57] reported that essential oils improved VH and CD, which led to an enhanced broiler performance. The histological improvement observed in chickens fed a diet containing essential oils can be explained by the reduction of toxins in the intestine due to the role of essential oils in modifying the microbial content of the intestine [55], which increases the absorption of nutrients due to the improvement in the histological structure of the intestine [58].

## Conclusions

In conclusion, our results proved that feeding broiler chickens a diet containing essential oils was able to mitigate the harmful effects of high ambient temperature (hot climate). The addition of lemon or garlic essential oil in a mixture or individually improved the growth performance, enhanced carcass characteristics, nutrient digestion, and blood lipid metabolism, and stimulated the activity of digestive enzymes and antioxidants. It also had an important role in improving intestinal health as an antimicrobial and a booster for histology.

## Materials and methods

### Chicks, design, and diets

This study was conducted at Poultry Research Farm, Desert Research Center, Egypt. A total of 480 broiler chicks (Ross 308) at 1-day-old with an average body weight of 40.3 g were randomized into four experimental groups of 120 chicks in six replicates (20 chicks/replicate). Chicks were raised during the summer season (hot climate) and under the same conditions in cages (30 × 35 × 40 cm), with free access to feed and water during the experimental period (35 days). The control (CON) group received a basal diet without supplements; the other three groups were as follows: a basal diet supplemented with (200 mg/kg diet) garlic essential oil (GEO), a basal diet supplemented with (200 mg/kg diet) lemon essential oil (LEO), and their mixture group (GLO) at 200 mg/kg feed, respectively. The basal diet formulated based on the nutritional requirements of the NRC [59] for broiler chicks is summarized in Table 7. The GEO and LEO were purchased from EL-Masrayia Company for the extraction of natural oils, in Cairo, Egypt. Commercial chicks (Ross 308) were raised at a temperature of 33°C and relative humidity of 65% until the third day, and then the chicks were raised with natural conditions (heat and humidity). Temperature and humidity were recorded twice a day (2 am and 2 pm); the relative humidity was 46% and the average temperature was 32.3 ℃ (inside the experimental chamber). We followed an immunization program according to the epidemiological conditions in Egypt, and it was as follows: at 1, 11, and 22 days of age; birds were vaccinated against Newcastle disease (ND), and at 15 days of age; birds were vaccinated against infectious bursal disease, as well injecting birds with influenza (H9N1) at 7 days of age.

**Table 7:** Ingredients and feed composition of broiler chickens (as fed-basis)

Item	Starter	Grower	Finisher
	(0-14d)	(15-28d)	(29-35d)
Corn	54.80	59.78	65.08
Soybean meal	34.60	29.20	23.60
Corn gluten meal	5.00	5.00	5.00
Soybean oil	1.65	1.70	2.00
Dicalcium phosphate	1.80	2.00	2.00
Calcium carbonate	1.30	1.40	1.40
Hcl Lysine	0.15	0.20	0.20
DL-Methionine	0.15	0.17	0.17
Salt	0.25	0.25	0.25
Mineral mix^a^	0.15	0.15	0.15
Vitamin mix^b^	0.15	0.15	0.15
Total	100	100	100
Calculated composition^c^			
ME (kcal/kg)	2950	3000	3100
Crude protein (%)	23	21	19
Ca (%)	1.011	1.070	1.063
Available P (%)	0.457	0.480	0.481

^a^Supplied per kilogram of diet: 4,000 IU of vitamin A; 80 mg of α-tocopherol acetate; 3 mg of vitamin K3; 2.5 mg of riboflavin; 2.5 mg of thiamin; 25 mg of nicotinic acid; 4 mg of pyridoxine; 800 mg of choline chloride; 0.3 mg of biotin; 1 mg of folic acid

^b^Supplied per kilogram of diet: 50 mg of Zn (zinc oxide); 20 mg of Fe; 60 mg of Mn; 12 mg of Cu (copper sulfate-pentahydrate); 0.30 mg of Cobalt; 0.35 mg of Se (sodium selenite); 0.55 g of Mg; 1.3 g of Na (sodium chloride); 0.45 mg of I (calcium iodate); in addition 10 mg of calcium pantothenate acid; 0.02 mg of coalmine

^c^Calculated according to NRC (1994); ME, Metabolizable energy

### Performance and Carcass indexes

The initial average weight was 40.7 g and the average weight of chicks from each experimental group was recorded. The feed intake was recorded weekly. The production performance indexes were estimated by calculating the average body weight (ABW), the average feed intake (AFI), the feed conversion rate (FCR), the European Production Efficiency Factor (EPEF), and the mortality rate. Carcass characteristics were calculated based on relative live BW (dressing, liver, and abdominal fat). The spleen, thymus, and bursa of Fabricius were weighed, as an index of the bird's immune status.

$$\mathrm{FCR}=\;the\mathit\;feed\mathit\;intake\mathit\;\mathit{\left(g\right)}\mathit/\mathit\;body\mathit\;weight\mathit\;gain\mathit\;\mathit{\left(g\right)}\mathit\;ratio$$

$$\mathrm{EPEF}=\left(\mathrm{Livability}\left(\%\right)\times\;BW\mathit\;\mathit{\left(g\right)}\right)/\left(age\mathit\;\mathit{\left({days}\right)}\mathit\times FCR\right)\times100$$

### Nutrient digestibility and Digestive enzymes

At the end of the experiment at the age of 35 days, five broilers were taken from each group (representing the average weight of each experimental group), and each bird was weighed and placed individually in the digestion cage and was starved for 12 hours to empty the digestive tract before the start of the digestion experiment. The excreta were collected twice a day (per 12 hours), after 3 days the excreta were weighed and dried (at 70°C for 24 hours) to estimate the necessary analyses. Dry matter (DM), crude protein (CP), and ether extract (EE) were estimated [60]. To detect the activity of digestive enzymes, 3 cm of ileum digesta of broiler chickens (5 birds/ group) were taken, and their contents were drained into sterile tubes, analyzed directly (amylase, trypsin, and lipase) as described by Najafi et al. [61].

### Plasma Constituents

At the end of the experiment at 35 days of age before slaughter, six blood samples were taken from each experimental group from the jugular vein in heparinized tubes and then centrifuged (2000×g for 10 min) to obtain plasma. Plasma cholesterol (total cholesterol, low-density lipoprotein (LDL), high-density lipoprotein (HDL)), triglycerides, total protein, albumin, creatinine, urea, aspartate aminotransferase (AST), and alanine aminotransferase (ALT) were determined via colorimetrically (Spectronic 1201, Milton Roy, Ivyland, PA, USA) according to the manufacturer’s instructions (Spinreact Co., Girona, Spain). Furthermore, globulin and albumin to globulin (A/G) ratios were calculated. The humoral immune response was measured as antibody titer against Newcastle disease virus (NDV) and avian influenza virus (AIV) by the hemagglutination inhibition (HI) test as described by Allan and Gough. [62]. The indications of the oxidation status in plasma were examined such as malondialdehyde (MDA), superoxide dismutase (SOD), and glutathione peroxidase (GPx) were performed using commercial kits (Spinreact Co. Girona, Spain).

### Microbial count and Intestinal Development

Immediately after euthanasia, 5 ileum samples (about 3 cm) from each treatment group were to estimate the histological characteristics of the ileum. The sample was fixed in a solution of 10% formalin, then placed on paraffin wax, and cut to be placed on glass slides for examination under a light microscope (ZEISS Axio Imager A2, Germany) as described by Abdel-Moneim et al. [3]. Villus height (VH) and crypt depth (CD) were measured, and VH: CD was calculated.

During slaughter, 5 grams of cecal were taken from 20 birds (5 per group) and placed in sterile bags, then made the necessary dilutions, then a mixture was taken from each sample and divided on Petri dishes to estimate the populations of the microbes under study as described by Elbaz [22]. Each microbe was incubated in its required environment, temperature, and time. The bacteriological examinations of the cecum digesta samples included total *lactic acid bacteria, Lactobacillus*, and *Escherichia coli* (MRS agar, MacConkey agar, and deMan agar, respectively). The number of microorganisms was converted to log10.

### Statistical analysis

Data experimental were analyzed by one-way ANOVA analysis using the General linear model (GLM) program of SAS [63] in a completely randomized design. Shapiro–Wilk, and Levenentests were used to test the normal distribution of data as well as the homogeneity of variance. Analyzing differences between groups was evaluated by Duncan’s multiple comparisons [64]. *P*-values < 0.05 are considered statistically significant.

## Data Availability

The datasets generated and analyzed during the current study are not publicly available. However, the corresponding author can provide it upon reasonable request after authorization and approval from Desert Research Center according to its policy.

## Abbreviations

ABW: Average body weight
AFI: Average Feed intake
AIV: Avian influenza virus
ALT: Alanine aminotransferase
AST: Aspartate aminotransferase
CD: Crypt depth
CON: Control basal diet
CP: Crude protein
DM: Dry matter
EE: Ether extract
EO: Essential oil
FCR: Feed conversion ratio
GEO: Basal diet with garlic essential oil
GLO: Basal diet with garlic and lemon essential oil
GPx: Glutathione peroxidase
HDL: High-density lipoprotein
LDL: Low-density lipoprotein
LEO: Basal diet with lemon essential oil
MDA: Malondialdehyde
NDV: Newcastle disease virus
SOD: Superoxide dismutase
VH: Villus height

## References

[1] -Kim HK (2016). Garlic supplementation ameliorates UV-induced photoaging in hairless mice by regulating antioxidative activity and MMPs expression. Molecules.

[2] -Elbaz AM, Ibrahim NS, Shehata AM, Mohamed NG, Abdel-Moneim AM (2021). Impact of multi-strain probiotic, citric acid, garlic powder or their combinations on performance, ileal histomorphometry, microbial enumeration and humoral immunity of broiler chickens. Trop Anim Health Prod.

[3] -Abdel-Moneim AM, Elbaz AM, Khidr RE, Badri FB (2020). Effect of in ovo inoculation of Bifidobacterium spp. on growth performance, thyroid activity, ileum histomorphometry, and microbial enumeration of broilers. Probiotics and antimicrobial proteins.

[4] -Choudhary M, Kochhar A, Sangha J (2014). Hypoglycemic and hypolipidemic effect of Aloe vera L. in non-insulin dependent diabetics. J Food Sci Technol.

[5] -Alagawany M, El-Saadony MT, Elnesr SS, Farahat M, Attia G, Madkour M, Reda FM (2021). Use of lemongrass essential oil as a feed additive in quail's nutrition: its effect on growth, carcass, blood biochemistry, antioxidant and immunological indices, digestive enzymes and intestinal microbiota. Poult Sci.

[6] -Jang HJ, Lee HJ, Yoon DK, Ji DS, Kim JH, Lee CH (2018). Antioxidant and antimicrobial activities of fresh garlic and aged garlic by-products extracted with different solvents. Food Sci Biotechnol.

[7] -Puvača N, Kostadinović L, Ljubojević D, Lukač D, Lević J, Popović S, Novakov N, Vidović B, Đuragić O (2015). Effect of garlic, black pepper and hot red pepper on productive performances and blood lipid profile of broiler chickens. Eur Poult Sci.

[8] -Bachiega TF, Sforcin JM (2011). Lemongrass and citral effect on cytokines production by murine macrophages. J Ethnopharmacol.

[9] -Prabuseenivasan S, Jayakumar M, Ignacimuthu S (2006). In vitro antibacterial activity of some plant essential oils. BMC Complement Altern Med.

[10] -Hong JC, Steiner T, Aufy A, Lien TF (2012). Effects of supplemental essential oil on growth performance, lipid metabolites and immunity, intestinal characteristics, microbiota and carcass traits in broilers. Livest Sci.

[11] -Hosseini SA, Nasari M, Zarai A, Lotfollahian H, Riyazi SR, Meimandipour A (2013). Effects of lemon essential oil on gastrointestinal tract, blood parameter and immune responses in broilers. Annals of Biological Research.

[12] -Al-Hijazeen M, Lee EJ, Mendonca A, Ahn DU (2016). Effect of oregano essential oil (Origanum vulgare subsp. hirtum) on the storage stability and quality parameters of ground chicken breast meat. Antioxidants.

[13] -Gopi M, Karthik K, Manjunathachar HV, Tamilmahan P, Kesavan M, Dashprakash M, Balaraju BL, Purushothaman MR (2014). Essential oils as a feed additive in poultry nutrition. Adv Anim Vet Sci.

[14] -Sohail MU, Hume ME, Byrd JA, Nisbet DJ, Ijaz A, Sohail A, Shabbir MZ, Rehman H (2012). Effect of supplementation of prebiotic mannan-oligosaccharides and probiotic mixture on growth performance of broilers subjected to chronic heat stress. Poult Sci.

[15] -Shakeri A, Zirak MR, Sahebkar A (2018). Ellagic acid: a logical lead for drug development?. Curr Pharm Design.

[16] Tekce E, Gül M. Effects of Origanum syriacum essential oil added in different levels to the diet of broilers under heat stress on performance and intestinal histology. Eur Poult Sci. 2016;80. 10.1399/eps.2016.157.

[17] -Patra JK, Das G, Bose S, Banerjee S, Vishnuprasad CN, del Pilar Rodriguez-Torres M, Shin HS (2020). Star anise (Illicium verum): Chemical compounds, antiviral properties, and clinical relevance. Phytother Res.

[18] -Saleh N, Allam T, El-Latif AA, Ghazy E (2014). The effects of dietary supplementation of different levels of thyme (Thymus vulgaris) and ginger (Zingiber officinale) essential oils on performance, hematological, biochemical and immunological parameters of broiler chickens. Global Vet.

[19] -Ocak N, Erener G, Burak Ak F, Sungu M, Altop A, Ozmen A (2008). Performance of broilers fed diets supplemented with dry peppermint (Mentha piperita L.) or thyme (Thymus vulgaris L.) leaves as growth promoter source. Czech J Anim Sci.

[20] -Alcicek AH, Bozkurt M, Çabuk M (2003). The effect of an essential oil combination derived from selected herbs growing wild in Turkey on broiler performance. South Afr J Anim Sci.

[21] Issa KJ, Omar JA (2012). Effect of garlic powder on performance and lipid profile of broilers.

[22] -Elbaz AM (2021). Effects of the diet containing fermented canola meal on performance, blood parameters, and gut health of broiler chickens. J Worlds Poult Res.

[23] Lippens M (2003). The influence of feed control on the growth pattern and production parameters of broiler chickens (Doctoral dissertation, Ghent University).

[24] -Cross AJ, Leitzmann MF, Gail MH, Hollenbeck AR, Schatzkin A, Sinha R (2007). A prospective study of red and processed meat intake in relation to cancer risk. PLoS Med.

[25] -Su G, Zhou X, Wang Y, Chen D, Chen G, Li Y, He J (2020). Dietary supplementation of plant essential oil improves growth performance, intestinal morphology and health in weaned pigs. J Anim Physiol Anim Nutr.

[26] -Xu YT, Liu LI, Long SF, Pan L, Piao XS (2018). Effect of organic acids and essential oils on performance, intestinal health and digestive enzyme activities of weaned pigs. Anim Feed Sci Technol.

[27] -Kırkpınar F, Ünlü HB, Özdemir G (2011). Effects of oregano and garlic essential oils on performance, carcase, organ and blood characteristics and intestinal microflora of broilers. Livest Sci.

[28] -Zeng Z, Zhang S, Wang H, Piao X (2015). Essential oil and aromatic plants as feed additives in non-ruminant nutrition: a review. J Anim Sci Biotechnol.

[29] -Prasad R, Rose MK, Virmani M, Garg SL, Puri JP (2009). Lipid profile of chicken (Gallus domesticus) in response to dietary supplementation of garlic (Allium sativum). Int J Poult Sci.

[30] Kim BC, Ryu YC, Cho YJ, Rhee MS (2006). Influence of dietary α-tocopheryl acetate supplementation on cholesterol oxidation in retail packed chicken meat during refrigerated storage. Bioscience, biotechnology, and biochemistry.

[31] Mundim AV, da Silva Martins JM, Marchini CFP, Guimarães EC, Gotardo LRM, Rinaldi FP, Bueno JPR, de M Nascimento, MRB and de Sousa GMR. Effect of age and cyclical heat stress on the serum biochemical profile of broiler chickens. Semina: Ciências Agrárias. 2017; 38(3), pp.1383–1392. DOI: 10.5433/1679-0359.2017v38n3p1383.

[32] -Alagawany M, El-Saadony MT, Elnesr SS, Farahat M, Attia G, Madkour M, Reda FM (2021). Use of lemongrass essential oil as a feed additive in quail's nutrition: its effect on growth, carcass, blood biochemistry, antioxidant and immunological indices, digestive enzymes and intestinal microbiota. Poult Sci.

[33] -Gibson C, Akinsoyinu Akintunde O, Tayo Grace O, Akinboye Olufunso E, Afodu Osagie J, Ndubuisi-Ogbonna Lois C, Ogbonnaya Faith C (2017). Serum biochemistry and sensory evaluation of broiler chicken fed cymbopogon citratus leaf meal. World.

[34] -Tiwari M, Dwivedi UN, Kakkar P (2010). Suppression of oxidative stress and pro-inflammatory mediators by Cymbopogon citratus D. Stapf extract in lipopolysaccharide stimulated murine alveolar macrophages. Food Chem Toxicol.

[35] Park SO, Hwangbo J, Ryu CM, Park BS, Chae HS, Choi HC, Kang HK, Seo OS, Choi YH. Effects of Extreme Heat Stress on Growth Performance, Lymphoid Organ, IgG and Cecum Microflora of Broiler Chickens. International Journal of Agriculture & Biology. 2013;15(6). https://www.fspublishers.org/published_papers/75376_..pdf

[36] Liang C, Xie XZ, Zhou YW, Jiang YY, Xie LJ, Chen Z. Effects of γ-aminobutyric acid on the thymus tissue structure, antioxidant activity, cell apoptosis, and cytokine levels in chicks under heat stress. Czech J Anim Sci. 2016;28(12):539–50. 10.17221/67/2015-CJAS. 61).

[37] -Aschbacher K, O’Donovan A, Wolkowitz OM, Dhabhar FS, Su Y, Epel E (2013). Good stress, bad stress and oxidative stress: insights from anticipatory cortisol reactivity. Psychoneuroendocrinology.

[38] -Zhang T, Xie J, Zhang M, Fu N, Zhang Y (2016). Effect of a potential probiotics Lactococcus garvieae B301 on the growth performance, immune parameters and caecum microflora of broiler chickens. J Anim Physiol Anim Nutr.

[39] -Awaad MHH, Elmenawey M, Ahmed KA (2014). Effect of a specific combination of carvacrol, cinnamaldehyde, and capsicum oleoresin on the growth performance, carcass quality and gut integrity of broiler chickens. Vet World.

[40] -Hanieh H, Narabara K, Piao M, Gerile C, Abe A, Kondo Y (2010). Modulatory effects of two levels of dietary Alliums on immune response and certain immunological variables, following immunization, in White Leghorn chickens. Anim Sci J.

[41] -Al-Sagheer AA, Mahmoud HK, Reda FM, Mahgoub SA, Ayyat MS (2018). Supplementation of diets for Oreochromis niloticus with essential oil extracts from lemongrass (Cymbopogon citratus) and geranium (Pelargonium graveolens) and effects on growth, intestinal microbiota, antioxidant and immune activities. Aquacult Nutr.

[42] Nidaullah H, Durrani FR, Ahmad S, Jan IU, Gul S (2010). Aqueous extract from different medicinal plants as anticoccidial, growth promotive and immunostimulant in broilers. J Agric Biol Sci.

[43] -Harikrishnan R, Kim MC, Kim JS, Balasundaram C, Heo MS (2011). Probiotics and herbal mixtures enhance the growth, blood constituents, and nonspecific immune response in Paralichthys olivaceus against Streptococcus parauberis. Fish Shellfish Immunol.

[44] -Bosetti GE, Griebler L, Aniecevski E, Facchi CS, Baggio C, Rossatto G, Leite F, Valentini FDA, Santo AD, Pagnussatt H (2020). Microencapsulated carvacrol and cinnamaldehyde replace growth-promoting antibiotics: effect on performance and meat quality in broiler chickens. An Acad Bras Cienc.

[45] Ojo OO, Kabutu FR, Bello M, Babayo U. Inhibition of paracetamol-induced oxidative stress in rats by extracts of lemongrass (Cymbropogon citratus) and green tea (Camellia sinensis) in rats. African Journal of Biotechnology. 2006;5(12).

[46] -Elwan HA, Elnesr SS, Mohany M, Al-Rejaie SS (2019). The effects of dietary tomato powder (Solanum lycopersicum L.) supplementation on the haematological, immunological, serum biochemical and antioxidant parameters of growing rabbits. J Anim Physiol Anim Nutr.

[47] -Ismail IE, Alagawany M, Taha AE, Puvača N, Laudadio V, Tufarelli V (2021). Effect of dietary supplementation of garlic powder and phenyl acetic acid on productive performance, blood haematology, immunity and antioxidant status of broiler chickens. Anim Bioscience.

[48] -Chen CH, Chan HC, Chu YT, Ho HY, Chen PY, Lee TH, Lee CK (2009). Antioxidant activity of some plant extracts towards xanthine oxidase, lipoxygenase and tyrosinase. Molecules.

[49] -Lin WC, Lee MT, Chang SC, Chang YL, Shih CH, Yu B, Lee TT (2017). Effects of mulberry leaves on production performance and the potential modulation of antioxidative status in laying hens. Poult Sci.

[50] -Zhu L, Liao R, Wu N, Zhu G, Yang C (2019). Heat stress mediates changes in fecal microbiome and functional pathways of laying hens. Appl Microbiol Biotechnol.

[51] -Wang XJ, Feng JH, Zhang MH, Li XM, Ma DD, Chang SS (2018). Effects of high ambient temperature on the community structure and composition of ileal microbiome of broilers. Poult Sci.

[52] -Kers JG, Velkers FC, Fischer EA, Hermes GD, Stegeman JA, Smidt H (2018). Host and environmental factors affecting the intestinal microbiota in chickens. Front Microbiol.

[53] -Xing L, Zhang H, Qi R, Tsao R, Mine Y (2019). Recent advances in the understanding of the health benefits and molecular mechanisms associated with green tea polyphenols. J Agric Food Chem.

[54] -Tiihonen K, Kettunen H, Bento MH, Saarinen M, Lahtinen S, Ouwehand AC, Schulze H, Rautonen N (2010). The effect of feeding essential oils on broiler performance and gut microbiota. Br Poult Sci.

[55] -Park JH, Kim IH (2018). Effects of a protease and essential oils on growth performance, blood cell profiles, nutrient retention, ileal microbiota, excreta gas emission, and breast meat quality in broiler chicks. Poult Sci.

[56] -Su G, Zhou X, Wang Y, Chen D, Chen G, Li Y, He J (2018). Effects of plant essential oil supplementation on growth performance, immune function and antioxidant activities in weaned pigs. Lipids Health Dis.

[57] -Alagawany MM, Farag MR, Kuldeep D (2015). Nutritional and biological effects of turmeric (Curcuma longa) supplementation on performance, serum biochemical parameters and oxidative status of broiler chicks exposed to endosulfan in the diets. Asian J Anim Veterinary Adv.

[58] -Wang S, Zhu S, Zhang J, Li H, Yang D, Huang S, Wei Z, Liang X, Wang Z (2020). Supplementation with yeast culture improves the integrity of intestinal tight junction proteins via NOD1/NF-κB P65 pathway in weaned piglets and H2O2-challenged IPEC-J2 cells. J Funct Foods.

[59] NRC, editor. Nutrient Requirements of Poultry. 9th rev. DC: National Academy Press, Washington; 1994.

[60] -AOAC (2000). Official methods of analysis of AOAC.

[61] Najafi MF, Deobagkar DN, Mehrvarz M, Deobagkar DD. Enzymatic properties of a novel highly active and chelator resistant protease from a Pseudomonas aeruginosa PD100. Enzyme and microbial technology. 2006;3;39(7):1433–40. 10.1016/j.enzmictec.2006.03.031.

[62] -Allan WH, Gough RE (1974). A standard haemagglutination inhibition test for Newcastle disease.(2) Vaccination and challenge. Vet Rec.

[63] SAS (2004). User’s Guide: Statics. Release 9.1.

[64] -Duncan DB (1955). Multiple ranges and multiple F tests. Biometrics.

